# A blood‐brain barrier‐like vascular gate in neuroendocrine cancers opens new avenues for immunotherapy

**DOI:** 10.1002/ctm2.70807

**Published:** 2026-09-02

**Authors:** Andrew Y. Chen, Yiyun Wang

**Affiliations:** ^1^ School of Life Sciences & School of Foreign Languages Peking University Beijing China; ^2^ Department of Biotherapy, Cancer Centre, State Key Laboratory of Biotherapy, West China Hospital Sichuan University Chengdu China

1

Immune checkpoint blockade (ICB) has emerged as a major pillar of modern cancer treatment. The basic principle of ICB is to release the “brakes” on the immune system, such as the PD‐1/PD‐L1 and CTLA‐4 pathways, thereby enabling T cells to attack tumors. A prerequisite for successful immunotherapy is the presence of sufficient numbers of T cells within the tumor microenvironment, as well as their direct physical access to tumor cells. Both T cells already present within tumors before treatment and those recruited during therapy have been shown to play critical roles in tumor killing.^1^, ^2^ Therefore, efficient recruitment of T cells from the circulation into tumors is essential. Tumors that fail to recruit T cells are classified as “cold” tumors and generally respond poorly to immunotherapy. However, the mechanisms underlying the formation of cold tumors remain largely unclear.

In our recent study, we identified a blood‐brain barrier‐like vascular gate (BVG) in small cell lung cancer (SCLC), a prototypical cold tumor.^3^ Paradoxically, SCLC often carries a high tumor mutation burden but is characterised by low levels of T cell infiltration and derives only modest clinical benefit from ICB. The BVG consists of endothelial tight junctions, a thickened basement membrane, and dense pericyte coverage. Together, these features form a highly restrictive vascular barrier that resembles the blood‐brain barrier (BBB) and blocks entry of immune cells from the circulation into tumor tissue. The BVG therefore provides a structural explanation for how SCLC can remain immunologically cold despite its high mutational burden.

Barrier‐like tumor vessels are not entirely unprecedented. In brain tumors, the blood‐tumor barrier (BTB) is thought to arise from the pre‐existing BBB. In contrast, the BVG is actively induced by SCLC cells themselves through the ASCL1‐IGFBP5‐IGF1R axis. ASCL1 induces IGFBP5 expression in tumor cells, and tumor‐derived IGFBP5 promotes IGF1‐IGF1R signalling in neighbouring endothelial cells to drive BVG formation. Disruption of ASCL1 or IGFBP5 in tumor cells, or of downstream IGF1R signalling in endothelial cells, compromises the BVG and increases T cell infiltration. Conversely, expression of ASCL1 or IGFBP5 is sufficient to induce BVG‐like structures in tumor models that normally lack them. Together, these findings define a tumor‐to‐endothelium signalling pathway through which neuroendocrine tumor cells establish a vascular barrier to immune‐cell entry.

Defining this pathway also creates a direct therapeutic opportunity. Genetic loss of *IGFBP5* disrupts the BVG and increases CD8^+^ T cell infiltration, providing proof of principle that the barrier can be reversed. More importantly, linsitinib (OSI‐906), an IGF1R inhibitor with established clinical safety, had previously shown little benefit as a single agent in SCLC. In the present study, OSI‐906 reduced BVG features and, when combined with anti‐PD‐1, markedly suppressed SCLC growth and increased CD8^+^ T cell infiltration in mouse models. These results suggest a new role for IGF1R inhibition—not as a direct antitumor strategy on its own, but as a means to improve immune access and sensitise tumors to checkpoint blockade (Figure 1).

**FIGURE 1:**
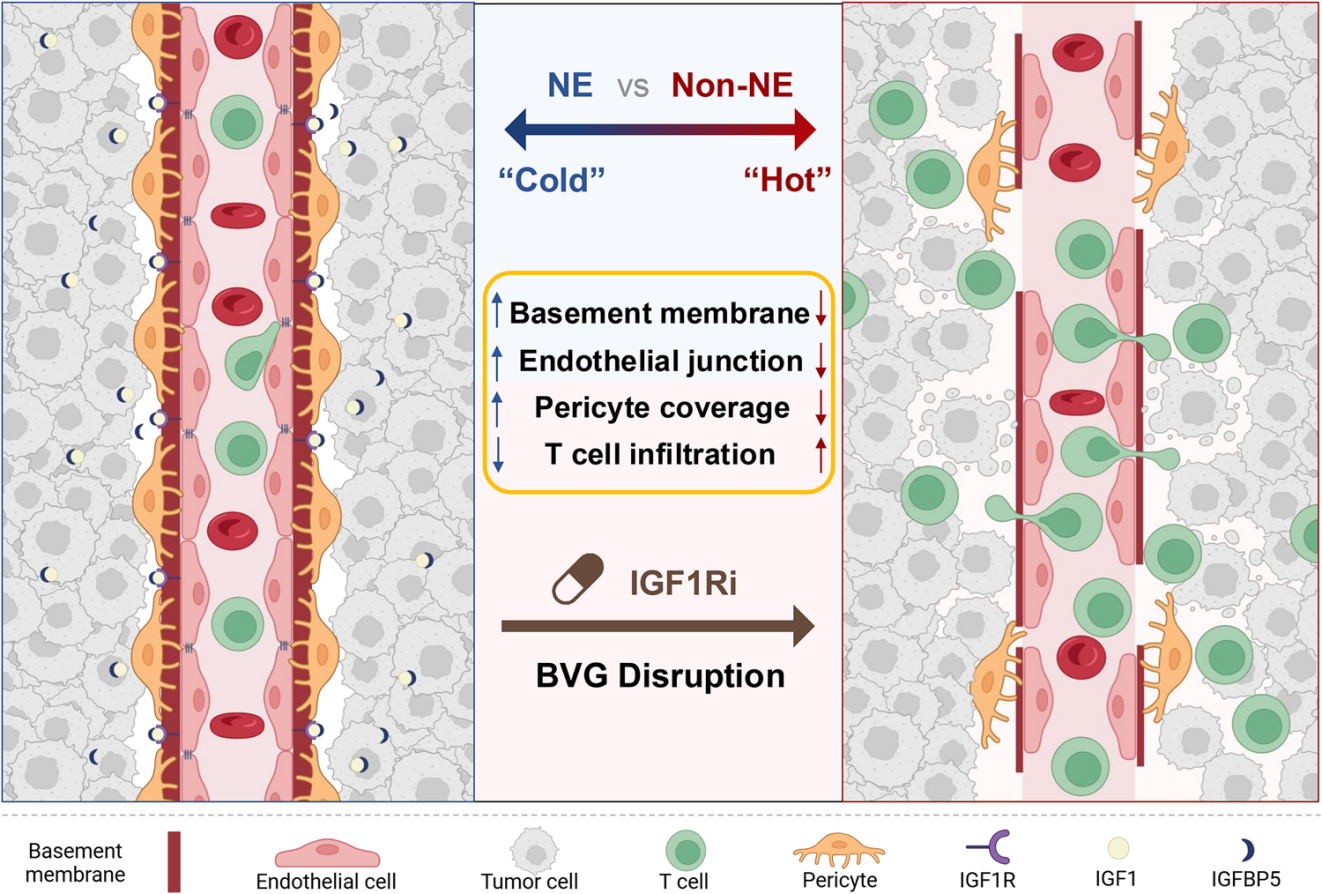
Disruption of the blood‐brain barrier‐like vascular gate (BVG) by IGF1R inhibition. The BVG restricts T cell infiltration through a thickened basement membrane, reinforced endothelial junctions, and dense pericyte coverage. IGF1R inhibition disrupts these barrier features and promotes T cell infiltration.

These therapeutic findings also broaden the concept of vascular normalization. Conventional vascular normalization mainly addresses the disorganised and excessively permeable vessels found in many solid tumors by reducing abnormal leakiness and improving vascular function. The BVG presents the opposite problem: excessive barrier function limits immune‐cell entry. In neuroendocrine cancers, effective vascular intervention may therefore require a controlled increase in permeability to permit T cell trafficking. This context‐dependent view suggests that vascular therapy should correct the specific vascular abnormality that limits treatment rather than apply the same normalization strategy to every tumor.

The significance of the BVG may extend beyond SCLC. BVG‐like structures and the associated ASCL1‐IGFBP5‐IGF1R program were also observed across several neuroendocrine cancers, supporting a broader role for this mechanism in neuroendocrine malignancies. The study also raises a wider possibility for other cold tumors: poor response to ICB may, in some settings, reflect restricted vascular access rather than insufficient T cell activation alone. Further functional validation in additional neuroendocrine cancers and other immune‐excluded tumors will be important to define how broadly this principle applies and whether vascular features can guide combination therapy. These findings therefore raise the possibility that vascular barriers could serve both as markers of immune exclusion and as therapeutic targets in a broader range of cold tumors.

Vascular entry, however, is only the first step in effective antitumor immunity. After crossing the vessel wall, T cells must still move through tumor tissue, establish productive contact with cancer cells and execute cytotoxic killing. Our previous work identified collagen‐rich barriers that restrict the intratumoral access of T cells to cancer cells.^4^ Resistance can persist even after T cells reach their targets: tumor‐derived *N*‐acetylaspartate can impair immunological synapse formation,^5^ while tumor‐intrinsic autophagy and broader genetic programs can reduce susceptibility to cytotoxic T cell‐mediated killing.^6^, ^7^ The BVG therefore defines an upstream checkpoint in a multistep process of antitumor immunity and may act together with downstream barriers that limit tumor‐cell access, productive immunological synapse formation and cytotoxic killing. Combining strategies that restore vascular entry with approaches that overcome these downstream barriers may therefore provide a more complete framework for improving immunotherapy efficacy.

## AUTHOR CONTRIBUTIONS

Andrew Y. Chen drafted the manuscript. Andrew Y. Chen and Yiyun Wang conceived the study and revised the manuscript.

## CONFLICT OF INTEREST STATEMENT

The authors declare no conflict of interest.
